# Further studies on the phlebotomine sandflies of the kala-azar endemic lowlands of Humera-Metema (north-west Ethiopia) with observations on their natural blood meal sources

**DOI:** 10.1186/1756-3305-3-6

**Published:** 2010-02-03

**Authors:** Teshome Gebre-Michael, Meshesha Balkew, Nega Berhe, Asrat Hailu, Yalemtsehay Mekonnen

**Affiliations:** 1 Aklilu Lemma Institute of Pathobiology, Addis Ababa University, PO Box 1176, Addis Ababa, Ethiopia; 2 Department of Microbiology, Immunology & Parasitology, Faculty of Medicine, Addis Ababa University, PO Box 1176, Addis Ababa, Ethiopia; 3 Department of Biology, Faculty of Science, Addis Ababa University, PO Box 1176, Addis Ababa, Ethiopia

## Abstract

**Background:**

Visceral leishmaniasis (VL) has been known to exist in northwest Ethiopia (Humera-Metema lowlands) since the early 1970s associated with large scale agricultural development activities, often resulting in outbreaks. The latest outbreak of the disease that has started around 1995 in both regions, has led to the present preliminary entomological surveys (1996-2005) the results of which are reported here. Sandflies were collected using CDC light traps and *Phlebotomus *females were dissected for *Leishmania *detection and isolation; freshly fed *Phlebotomus *females collected were subsequently tested for their blood meal sources using ELISA. All *Phlebotomus *collections were identified to species.

**Results:**

During the surveys (1996-2005), a total of 1963 sandflies of six *Phlebotomus *species (*P. orientalis*, *P. papatasi*, *P. bergeroti, P. duboscqi*, *P. rodhaini *and *P. alexandri*) were recorded from the study areas: the predominant species was *P. orientalis *in both localities. None of the total 618 *P. orientalis *females dissected (506 from Metema and 112 from Humera), nor the total 114 females of four other species dissected (*P. papatasi*, *P. duboscqi*, *P. bergeroti *and *P. rodhaini*) was infected with *Leishmania *promastigotes. ELISA-based blood meal analysis of 273 fresh fed *P. orientalis *females collected from Metema revealed a remarkably high bovine blood feeds (92%) with only 2.2% of human blood feeds.

**Conclusion:**

Based on abundance and other circumstantial evidences (its proven role in Sudan), *P. orientalis *is the most likely vector of VL in northwest Ethiopia, pending further clarifications. The zoophagic feeding behaviour of *P. orientalis *detected in the present study could have epidemiological significance, but more investigations are required in this and other behavioural characteristics towards appropriate management of the vector.

## Introduction

Visceral leishmaniasis (VL) has been known in the Humera-Metema lowlands (northwest Ethiopia) since at least 1970 [1] and remains endemic in the region[2]. In this region, the disease is particularly associated with migration of non-immune labourers from the surrounding highland regions to the extensive agricultural development schemes in the lowlands [3,4]. Thus, at various times in the past, several outbreaks of the disease have occurred in the region. The most recent one that has started in 1995 appeared to be more serious claiming the lives of about 100-200 temporary farm labourers mainly from Maykadra village in Humera in just five months (August-December), in addition to 56 cases that were treated [2]. As far as is known, detailed epidemiological investigation in the region is lacking to reveal the exact distribution and magnitude of the recent outbreak of the disease at the time, other than hospital-based reports of Médecins Sans Frontiéres-Holland (MSF) of 1300 patients having been treated in Humera Hospital in three years since December 1997 [5].

Following the earliest outbreaks of the disease in the 1970s [1,6], entomological studies conducted in the region [7,8] revealed the presence of four species of *Phlebotomus *(*P. oreintalis*, *P. papatasi*, *P. duboscqi *and *P. alexandri*). The authors also mentioned the performance of a limited but unsuccessful dissection of unspecified species and number. However, they postulated that *P. oreintalis *might be the vector of the disease in the region based on circumstantial evidence from the knowledge already available in the adjacent endemic regions of Sudan [9].

Considering the public health importance of the disease and the recent outbreak in the region, we carried out entomological surveillance to study the *Phlebotomus *species present and determine their leishmanial infection rate towards incriminating the vector (s). The host preferences of the *Phlebotomus *females were also determined as there was little or no information on the vectors of leishmaniasis here or elsewhere in Ethiopia.

## Materials and methods

### Study area

The investigation was carried out at various times from 1996 to 2005 in Humera and Metema districts. Humera and Metema are located about 370 and 165 kms north-west, and west of Gondar Town, repectively. The distance between the two major study areas is about 150-180 kms. In Humera, six settlement localities (Rawyan, Adebay, Maykadra, Woldeab and Humera Town) were included in the surveys; all lie between latitudes 14° 12'-14° 55'N and longitudes 36° 31'-36° 46'E within 28 kms from Humera Town. In Metema district, eight settlement areas (Metema Town, Gendawuha, Kumer, Aftit, Kokit, Gebgeb, Zingri and Mender 6 & 7) lying between latitudes of 12° 45'-12° 58'N and longitudes 36° 11'-36° 25'E within 37 kms from Metema Town were surveyed. The study localities in both districts (Humera and Metema) lie at altitudes 500-700 meters above sea level (masl) and have similar ecology where the previous dominant vegetation, *Acacia-Balanites *'forest' has now been immensely cleared for large scale agricultural production of cash crops (cotton, sesame, sorghum), and more recently and intensively for settlement(construction) and fire wood as well. Large number of cattle, sheep and goats are also raised in both regions. Annual rainfall ranges between 500-800 mm with the long rains from July to September [7] and with temperatures usually high (25-30°C) most of the year [10] reaching as high as 40°C during the months of April-May.

### Collection of sandflies

Sandflies were collected on two occasions in Humera district [25 March - 31 April 1996 and 14-20 January 1997) and on three occasions in Metema district (21-24 June 2003; 5-10 February 2005 and 28 March - 6 April 2005). They were collected using mainly CDC miniature light traps (John W. Hock, Gainesville, FL) but also sticky traps on a limited scale from outdoor situations and peri-domestic habitats. The outdoor trapping habitats included *Acacia*-*Balanites *forest, and farm fields, whereas, peridomestic habitats included near animal enclosures, compounds and homesteads. Traps were also occasionally set inside houses but were unproductive on most occasions. However, detailed studies based on long-term collection of sandflies for determination of seasonality, infection rates, habits and habitats were not done because of the long distance from the main laboratory and limited budget. Of the collection, *Phlebotomus *females were sorted out for dissection or blood meal analysis. Females with fresh blood meals in the collection were stored for later blood meal analysis (see below).

### Dissections of sandflies for age determination and natural infections

The ovaries of unfed *Phlebotomus *females were drawn out together with the guts to determine their parous state [11,12]. The guts of the parous females were immediately examined for presence of promastigotes under the microscope [11,12]. Similarly, after careful removal of the developing ova of each gravid and semi-gravid female, the gut was also examined microscopically for promastigote infection.

### Blood meal collection

During dissection, any freshly blood fed *Phlebotomus *female encountered was preserved for later blood meal analysis. For this purpose, the head of each fed female was carefully severed and slide-mounted for later species identification. The remaining body (thorax and abdomen) was individually placed in empty antibiotic capsules with silica gel grains and cotton pads inside, bearing a corresponding label with the mounted specimen on the slide. Once in the laboratory, they were stored at -20°C until blood meal analysis by ELISA.

### Mounting and Identification of sandflies

After dissection and examination for promastigote infection, and also collection of blood meals, the head and remaining body parts of each *Phlebotomus *female was separately slide-mounted in gum-chloral for later identification. In the field, undissected *Phlebotomus *females (inadvertently missed out during dissection) and males were directly preserved in 70% alcohol for later identification in the laboratory. In the laboratory, the alcohol preserved *Phlebotomus *sandflieswere initially rinsed in water, then cleared in Nesbitt's solution [13] for 12-24 hours and slide-mounted in Berlese's gum chloral under individual cover glasses. Identifications of the mounted specimens were based on keys [14,15] and other morphological criteria [16,17]. All *Sergentomyia *species in the collection were also preserved in alcohol, but most remain unidentified because of their high abundance and unimportance in leishmaniasis transmission.

### Blood meal analysis

The blood meals in the sandfly guts were screened for their sources of origin by direct ELISA [18]. Each blood meal was individually triturated in 2 ml Eppendorf tubes with micro-tissue grinders s (Kontes Scientific Glassware, Vineland, N.J.) to which 50 μl of 0.01 M phosphate buffered saline (PBS), pH 7.4 was added. Samples were then mixed with PBS to desired dilutions and kept in the refrigerator (4°C) until tested. Sandfly triturate (50 μl) was diluted in PBS (1:50) and 50 μl was added to wells of polyvinyl chloride, U-shaped, 96-well micro titer plates (Dynatech Laboratories, Inc., Alexandria, Va), which were covered and incubated at 4°C overnight. Each well was then washed twice with 200 μl PBS containing 0.5% Tween 20 (PBS-Tw 20). This was followed by the dilution of 50 μl host-specific conjugate (antihost IgG, H and L) diluted 1:2,000 in 0.5% boiled casein containing 0.025% Tween 20. The boiled casein was prepared by dissolving 5 g casein in 100 ml of 0.1 N NaOH by boiling, adding 900 ml PBS, adjusting pH to 7.4 adding 0.1 g Thimerosal (Sodium ethylmercurithiosalicylate) and 0.02 gm phenol red. After 1 h, wells were washed three times with 200 μl PBS Tween 20, and then 100 μl of ABTS (2, 2-azino-di- [3-ethyl benzthiazoline sulfonate]) peroxidase substrate (Kirkegaad and Perry Laboratories, Inc.) was added to each well. Absorbance at 414 nm was determined with an ELISA reader 30 min after the addition of substrate. Negative controls were prepared using unfed laboratory-reared female *P. orientalis *[19]. Positive serum controls were done by making host serum: PBS dilutions of 1:50 [18]. Samples were screened first for human and then for bovine blood on separate plates. Each plate contained a positive control of human or bovine; four negative controls and test samples (1:50 dilution in PBS for all cases). Test samples were considered positive if absorbance values exceeded the mean plus three times the standard deviation of four negative controls. Positive and negative control samples were tested on each micro titer plate as inter-plate variation for absorbance values of controls can be significant if plates are not read at consistent times following the addition of substrate.

## Results

During the studies, a total of 1963 sandflies of the genus *Phlebotomus *comprising six species (*P. orientalis*, *P. papatasi*, *P. bergeroti*, *P. duboscqi*, *P. rodhaini *and *P. alexandri*) were collected from the study areas (Table 1). The predominant species in both localities was *P. orientalis *followed by *P. papatasi *in Humera and *P. bergeroti *in Metema. However, Metema was more productive for *P. orientalis*. All the six species were recorded in Metema while only four species were recorded in Humera. A single specimen of *P. alexandri *was only collected from Metema.

**Table 1:** *Phlebotomus *species collected and dissected in Humera and Metema lowlands (northwest Ethiopia)

Species	Humera area		Metema area		Total		Total (M+F)
		
	M	F	M	F	M	F	
*P. (Larroussius) orientalis*							
No. collected	130	131	601	802	731	933	1664
No. dissected (unfed & gravid)	-	112	-	484	-	596	
*P. (Phlebotomus) papatasi*							
No. collected	96	109	1	1	97	110	207
No. Dissected	-	74	-	1	-	75	
*P. (Phlebotomus) bergeroti*							
No. collected	9	11	15	16	24	27	51
No. Dissected	-	11	-	14	-	25	
*P. (Phlebotomus) duboscqi*							
No. collected	2	4	7	3	9	7	16
No. Dissected	-	4	-	3	-	7	
*P. (Anaphlebotomus) rodhaini*							
No. collected	0	0	13	11	13	11	24
No. Dissected	-	-	-	7	-	7	
*P. (Paraphlebotomus) alexandri*							
No. Collected	0	0	1	0	1	0	1
Total collected	237	255	638	833	875	1088	1963
Total dissected	-	201	-	509	-	710	

Note:- M = Male; F = Female

The abdominal categories and parous rates of the different *Phlebotomus *species detected during dissection in the two major study areas are shown in Table 2. Of the total 802 female *P. orientalis *collected in Metema, 334 (41.6%) were hungry ('unfed'), 281 (35.04%) were freshly engorged and 172 (21.4%) were gravid and semi-gravid females. Of the 334 unfed *P. orientalis *females dissected for parous state (parous or nulliparous), 115 (34.4%) were parous; the remaining 15 specimens were missed out during dissection, and were only identified after mounting of the alcohol preserved specimen. Likewise, of the total 115 female *P. orientalis *collected in Humera, 54 (47%) were unfed, 58 (50.4%) were gravid and 3 (2.6%) were fresh fed females. Of the 54 'unfed' *P. orientalis *females dissected for the ovarian state, 16 (30.2%) were parous.

**Table 2:** Abdominal status of female *Phlebotomus *species determined during dissection.

Species	No. Collected*		No. fresh feds		No. gravid		No. unfed		No. parous (%)**	
	
	Humera	Metema	Humera	Metema	Humera	Metema	Humera	Metema	Humera	Metema
*P. orientalis*	115	802	3	281	58	172	54	334	16 (30.2)	115 (34.4%)
*P. papatasi*	109	1	0	0	29	0	44	1	21 (47.7)	0
*P. bergeroti*	11	16	1	2	5	10	4	1	2 (50)	0
*P. duboscqi*	4	3	0	0	0	2	4	0	1 (25)	0
*P. rodhaini*	0	13	0	0	0	1	0	8	0	2
*P. alexandri*	0	0	0	0	0	0	0	0	0	0

* Includes also the undissected female sandflies (15 *P. orientalis *from Metmea were missed out during dissection in the field)

**Based on dissection of the unfed female sandflies

The results of dissection from both localities revealed no leishmanial infection in 618 (unfed and gravid) females of *P. orientalis *dissected (112 from Humera and 506 from Metema), 75 *P. papatasi *(74 from Humera and one from Metema), 25 *P. bergeroti *(11 from Humera and 14 from Metema), 7 *P. duboscqi *(4 from Humera and 3 from Metema), and 7 *P. rodhaini *(all from Metema).

Of the total 281 freshly engorged female *P. orientalis*, collected from Metema area, 273 were analyzed for their blood meal sources; results are summarized in Table 3. The predominant of the samples [n = 250 (91.6%)] was positive for bovine blood origin, and only 6 (2.2%) (n = 6) were of human origin, and 7 (2.6%) were of mixed blood sources (bovine and human); the remaining 10 (3.7%) were unidentified. Fourteen blood meals (eight of *P. orientalis *from Metema, three from Humera, two *P. bergeroti *from Humera and Metema and one of *P. rodahini *from Metema) could not be analyzed as they could not be retrieved from the freezer.

**Table 3:** Bloodmeal identification for *Phlebotomus orientalis *from Metema area (2003 to 2005).

Village	No. tested	+ve for bovine blood (%)	+ve for human blood (%)	Mixed bovine & human blood	Unidentified
Kokit	237	220 (92.8)	6 (2.5)	5 (2.2)	6 (2.5)
Gendawuha	20	17 (85)	0	1 (5)	2 (10)
Gebgeb	13	12 (92.3)	0	1 (7.7)	0
Kumer	3	1 (33.3)	0	0	2 (66.7)

Total	273	250 (91.6)	6 (2.2)	7 (2.6)	10 (3.7)

## Discussion

In the present preliminary investigation, close to 2000 sand flies of six *Phlebotomus *species (*P. orientalis*, *P. papatasi*, *P. bergeroti*, *P. duboscqi*, *P. rodhaini *and *P. alexandri*) were collected from both major study areas in northwest Ethiopia. The former is the predominant species and is consistent with previous records in the region [7,8]. Ecologically, most of the Humera-Metema lowlands seem similar to the known VL endemic areas of the Sudan which are characterized by the presence of *Acacia *-*Balanites *forest and an expanse of deeply cracking 'black cotton-clay soil' (vertisols) where the vector *P. orientalis *is known to thrive best [9]. Much of the forest in NW Ethiopia, however, is now cleared for settlement and huge commercial farms.

The occurrence of three members of the subgenus *Phlebotomus *together (*P. papatasi*, *P. bergeroti *and *P. duboscqi*) in this part of Ethiopia is consistent with the map shown by Lewis [15] on the known distribution of the subgenus in the Old World. However, zoonotic cutaneous leishmaniais (*L. major*) is not yet known in this part of Ethiopia, but is known to be much more common in neighboring Sudan where, in the past, several outbreaks have been reported in various parts of the country including Khartoum in the 1980s [20]; *P. papatasi *was thought to be involved in the transmission. *Phlebotomus duboscqi *and *P. bergeroti *are sympatric in the Awash Valley (Rift Valley) of north-east Ethiopia [21], whereas, the former species appears to exist solely in much of the southern and southwestern Ethiopia [11,22,23] and Kenya where it is also involved in *L. major *transmission [24].

In the present limited study, no natural infection was detected in 618 *P. orientalis *females, nor in a little over 100 females of other species dissected (*P. papatasi*, *P. bergeroti*, *P. duboscqi*, and *P. rodhaini). Phlebotomus orientalis *however is highly likely to be the vector in northwest Ethiopia as previously suggested [7,8] as it was so in northern Ethiopia [25,26]. The parous rates of the dissected *P. orientalis *females were moderately low (35%) and is comparable with those observed for the same species in Addis Zemen, northern Ethiopia [26], Awash Valley, Eastern Ethiopia [Gebre-Michael and Balkew unpublished) and for *P. martini *and *P. celiae *in Aba Roba, southern Ethiopia [12]. This implies that a large number of sandflies sampled over a longer time, covering the different seasons of year will have to be dissected to detect natural infections as this would increase the chance of getting more parous and gravid/semi-gravid females that are potentially infected. PCR-based techniques could have detected *Leishmania *in the sandflies [27], but facilities were not available in our laboratory nor any prior arrangement was made with any laboratory abroad; we were simply confident to detect and isolate parasites by the classical method based on our previous experience [11,12,21].

The higher number of fed, gravid and semi-gravid females in the sandflies collected near hosts (in villages) and the proximity of engorged females to their blood meal sources, indicate that sandflies (*P. orientalis *and *P. papatasi*) usually remain near their hosts after feeding. A similar observation has also been made for *P. longipes*, one of the vectors of CL (*L. aethiopica*) in Ethiopia [28]. However, in CDC light traps set in the *Acacia *forest or farm fields further away from houses/compounds, very few blood fed females were collected despite a very high population density of sandflies (mostly *P. orientalis*) caught in the traps compared to those set near villages. This suggests that the majority of sandflies either migrates to villages for feeding on their preferred hosts or those feeding outdoors on wild animals are probably as widely dispersed as their wild hosts are, thus, becoming rare in the trap collections.

The results of blood meal analysis showing about 92% positive for bovine blood and only about 2% for human and 2.6% for mixed feeding (cattle & human) seem to suggest that *P. orientalis *strongly prefers bovines to humans for its blood meals. In some settlements of Metema, large number of cattle is usually kept in open or fenced enclosures next or close to houses (5-30 meters) with a group of adult men guarding the animals from theft during the whole night. Goats, sheep, donkeys, chicken and dogs are also present but these are usually smaller in numbers. Among wild animals, monkeys were occasionally seen on riverine vegetation and were far from human dwellings. Thus, it is not yet clear whether this bovine blood feeding behaviour of *P*. *orientalis *is due to a preference for cattle or due to the mere presence of an abundant population of cattle outdoors than humans which are normally confined indoors during the night except during the hot seasons. In some areas where *P*. *orientalis *has so far been observed to occur in Ethiopia, its association with cattle seems remarkable, although this has not been systematically studied in detail. For example, in the Awash Valley (Rift Valley) of north eastern Ethiopia where the Afar pastoralists keep huge herds of cattle, camels, goats and sheep, more freshly fed *P. orientalis *and *P. bergeroti *were caught in light traps set near animals and human settlements [Gebre-Michael and Balkew, unpublished]. A similar observation was also made in a VL endemic area of Libo Kemkem Distrtict in northern Ethiopia [Gebre-Michael and Balkew, unpublished]. Analysis of 94 blood meals of *P. orientalis *(collected from the Awash Valley) based on counter current immuno-electrophoresis (CCIE) technique, revealed 14 (15%) from cattle origin, 9 (9.6%) from camels, 51 (54.3%) from mixed sources, 2 from squirrel (2.1%), and only a single specimen (1.1%) of each human, mongoose and donkey [29]. The mixed blood sources came predominantly from cattle-camel hosts (60%) followed by camel-cattle-squirrel hosts (25%). Thus, in the Awash Valley, *P. orientalis *seems to be an opportunistic feeder, with some degree of preference to cattle and camels. Comparison of the present work with any previous works other than the Awash Valley was impossible, for there is no such similar study on the natural feeding preferences of *P. orientalis *elsewhere including the Sudan where it has been more studied. However, the role played by cattle as blood meal source for several sandfly species was highly indicated in various studies [28,30-34].

Despite the setting of traps near animals and human settlements, only a single blood meal was of human origin in Metema. Several explanations may be forwarded. One of these could be due to the fact that *P. orientalis *is essentially exophagic and rarely enters houses to feed on humans as collections were either nill or negligible in light traps set in houses. It is also possible that the smoke inside houses may drive off sandflies from entering houses, thus preventing man-sandfly contact. Thirdly, even when people sleep outdoors for looking after cattle and/or due to the hot weather conditions in the area, the clothing over the body at night may prevent or reduce man-sandfly contact. A final explanation may be that cattle were simply the preferred hosts due to their large size and number, despite the presence of other animals including humans in the area, as previously suggested by several investigators based on observations with other species of sandflies [32,35,36]. In contrast, some authors [37,38] observed host size not to be important, as smaller rodents and bats attracted more sandflies (*Lutzomyia *species) than larger animals (primates and carnivores). At any rate, multiple factors must come into play to determine the feeding behaviour of a given sandfly species and calls for detailed and systematic studies to elucidate the factors behind host selection in sandflies. For the present, the predilection of *P. orientalis *for cattle could have epidemiological significance in controlling the disease. This control is probably already in operation in nature by cattle serving as barriers reducing man-sandfly contact (zooprophylaxis). Similar phenomena have been observed in some endemic areas of leishmaniasis both in the Old World and New World, leading some investigators to propose the use of cattle for zooprophylaxis [31,32,39]. In addition, treatment of cattle with insecticides (ITC), pour-on insecticides, or insecticide impregnated collars for cattle ('dog collar' type: [40,41]) could be developed for use in the control of zoophagic sandflies. However, further and detailed studies on the feeding patterns of *P. orientalis *and other vectors of leishmaniasis in Ethiopia in different habitats with different mixtures of potential vertebrate hosts are needed to better understand their natural feeding behaviours and come up with reasonable conclusions.

## Competing interests

The authors declare that they have no competing interests.

## Authors' contributions

TGM, MB, NB and AH conceived the investigation; the former two performed the field and laboratory experiments, analyzed the data and prepared the manuscript. NB, AH and YM facilitated and participated in the investigation, read and improved the manuscript.

## References

[B1] Tekle A Neri P Debessai A Kala-azar in Humera (North-West Ethiopia) Parassitologia 1970 12 21 25

[B2] Hailu A Gebre-Michael T Berhe N Balkew M Berhane Y, Hailemariam D, Kloos H Leishmaniasis Epidemiology and Ecology of Health and Disease in Ethiopia 2006 Shama Books, Addis Ababa 615 634

[B3] Mengesha B Abuhoy M Kala-azar among labour immigrants in the Metema-Humera region of Ethiopia Trop Geog Med 1978 30 199 206 726033

[B4] Maru M Clinical and laboratory features and treatment of visceral leishmaniasis in hospitalized patients in northern Ethiopia Am J Trop Med Hyg 1979 21 15 18 10.4269/ajtmh.1979.28.15 219723

[B5] Ritmeijer K Veeken H Melaku Y Leal G Amsalu R Seaman J Davidson RN Ethiopian visceral leishmaniasis: generic and proprietary sodium stibogluconate are equivalent; HIV co-infected patients have poor outcome Trans R Soc Trop Med Hyg 2001 95 668 672 10.1016/S0035-9203(01)90110-5 11816442

[B6] Fuller GK Lemma A Haile T Atwood CA Kala-azar in Ethiopia. I. Leishmanin skin-test in Setit-Humera, a kala-azar endemic area in northwestern Ethiopia Ann Trop Med Parasitol 1976 70 147 163 938122 10.1080/00034983.1976.11687108

[B7] Gemetchu T Zerihune A Assefa G Lemma A Observations on the sandfly (Phlebotomidae) fauna of Setit-Humera (Northwestern Ethiopia) Ethiop Med J 1975 13 41 51 1227868

[B8] Gemetchu T The distribution of sandflies (Diptera, Psychodidae, Phlebotominae) in north-west Ethiopia Sinet: Ethiop J Sci 1983 6 65 73

[B9] Hoogstraal H Heyneman D Leishmaniasis in the Sudan Republic. 30. Final epidemiological report Am J Trop Med Hyg 1969 18 1091 1210

[B10] Gamachu D Aspects of Climate and Water Budget in Ethiopia 1997 Addis Ababa University Press, Addis Ababa, Ethiopia

[B11] Gebre-Michael T Pratlong F Lane RP *Phlebotomus (Phlebotomus) duboscqi *(Diptera: Phlebotominae), naturally infected with *Leishmania major *in southern Ethiopia Trans R Soc Trop Med Hyg 1993 87 10 11 10.1016/0035-9203(93)90399-B 8465376

[B12] Gebre-Michael Lane RP The roles of *Phlebotomus martini *and *P. celiae *(Diptera: Phlebotominae) as vectors of visceral leishmaniasis in the Aba Roba focus, southern Ethiopia Med Vet Entomol 1996 10 53 62 10.1111/j.1365-2915.1996.tb00082.x 8834743

[B13] Minter DM Three new sandflies (Diptera, Psychodidae) from East Africa, with notes on other species Bull Entomol Res 1963 54 483 495 10.1017/S0007485300048963

[B14] Abonnenc E Les phlébotomes de la région Ëthiopiene (Diptera, Psychodidae) Mém Off Rech Sci Tech Outre-Mer 1972 55 1 289

[B15] Lewis DJ A taxonomic review of the genus *Phlebotomus *(Diptera: Psychodidae) Bull Brit Mus (NH) (Ent) 1982 45 121 209

[B16] Lane RP Fritz G The differentiation of the leishmaniasis vector *Phlebotomus papatasi *and *P. bergeroti *(Diptera: Phlebotominae) Syst Entomol 1986 11 439 445 10.1111/j.1365-3113.1986.tb00535.x

[B17] Gebre-Michael T Medhin G Morphometric separation of the females of *Phlebotomus *(*Phlebotomus) duboscqi *and *P. (P.) bergeroti *(Diptera: Psychodidae) J Med Entomol 1997 34 383 386 9220670 10.1093/jmedent/34.4.383

[B18] Beier JC Perkins PV Wirtz RA Koros J Diggs D Gargan TP Koech DK Blood meal identification by direct Enzyme-Linked Immunosorbent Assay (ELISA), tested on *Anopheles *(Diptera: Culicidae) in Kenya J Med Entomol 1988 25 9 16 3357176 10.1093/jmedent/25.1.9

[B19] Ngumbi PM Lawyer PG Johnson RN Kiilu G Asiago C Identification of sandfly blood meals from Baringo district, Kenya, by direct enzyme-linked immunosorbent assays (ELISA) Med Vet Entomol 1992 6 385 388 10.1111/j.1365-2915.1992.tb00638.x 1463906

[B20] El Safi SH Peters W Studies on the leishmaniasis in the Sudan. 1. Epidemic of cutaneous leishmaniasis in Khartoum Trans R Soc Trop Med Hyg 1991 85 44 47 10.1016/0035-9203(91)90151-N 2068758

[B21] Gebre-Michael T Balkew M Ali A Ludovisi A Gramiccia M The isolation of *Leishmania tropica *and *L. aethiopica *from *Phlebotomus (Paraphlebotoms) *species (Diptera: Psychodidae) in the Awash Valley, northeastern Ethiopia Trans R Soc Trop Med Hyg 2004 98 64 70 10.1016/S0035-9203(03)00008-7 14702839

[B22] Balkew M Hailu A Berhe N Gemetchu T Leishmaniasis in the lower Omo plains, south-western Ethiopia: The sandfly fauna Ethiop Med J 1999 37 31 39

[B23] Balkew M Gebre-Michael T Berhe N Ali A Hailu A Leishmaniasis in the middle course of the Ethiopian Rift Valley: II Entomlogical Observations Ethiop Med J 2002 40 271 282 12602251

[B24] Beach R Kiilu G Kendricks L Oster C Leeuwenberg J Cutaneous leishmaniasis in Kenya. Transmission of *Leishmania major *to man by the bite of *Phlebotomus duboscqi* Trans R Soc Trop Med Hyg 1984 78 747 751 10.1016/0035-9203(84)90006-3 6533846

[B25] Ashford RW Hutchinson MP Bray RS Kala-azar in Ethiopia: Epidemiological investigations in a highland valley Ethiop Med J 1973 11 259 264 4803210

[B26] Gebre-Michael T Balkew M Alamirew T Gudeta N Reta M Preliminary entomological observations in a highland area of Amhara region, northern Ethiopia, with epidemic visceral leishmaniasis Ann Trop Med Parasitol 2007 101 367 370 10.1179/136485907X176382 17524252

[B27] Rossi E Bongiorno G Ciolli E Di Muccio T Scalone A Gramiccia M Gradoni L Maroli M Seasonal phenology, host-blood feeding preferences and natural *Leishmania *infection of *Phlobotomus perniciosus *(Diptera, Psychodidae) in a high-endemic focus of canine leishmaniasis in Rome province, Italy Acta Trop 2008 105 158 165 10.1016/j.actatropica.2007.10.005 18035329

[B28] Foster WA Boreham PFL Tempelis CH Studies on leishmaniasis in Ethiopia. IV. Feeding behaviour of *Phlebotomus longipes *(Diptera: Psychodidae) Ann Trop Med Parasitol 1972 66 433 443 4656445

[B29] Mamo H *Production of specific antisera against selected mammals and identification of blood meals of phlebotomine sandflies transmitting visceral leishmaniasis in Ethiopia* MSc thesis 1999 Addis Ababa University, Addis Ababa 49

[B30] Mukhopadhayay AA Chakravarty AK Bloodmeal preference of *Phlebotomus argentipes *and *Ph. papatasi *of north Bihar, India Indian J Med Res 1987 86 475 480 3127337

[B31] Ghosh KN Bhattacharya A Ghosh TN Blood meal analysis of *Phlebotomus argentipes *in eight districts of West Bengal J Com Dis 1990 22 67 71 2230024

[B32] Morrison AC Ferro C Tesh RB Host preferences of the sandfly *Lutzomyia longipaplpis *at an endemic focus of American visceral leishmaniasis in Colombia Am J Trop Med Hyg 1993 49 68 75 8352394 10.4269/ajtmh.1993.49.68

[B33] Guy MW Killick-Kendrick R Gill GS Rioux J-A Bray RS Ecology of leishmaniasis in the south of France 19. Determination of the hosts of *Phlebotomus ariasi *Tonnoir, 1921 in the Cevennes by blood meal analysis Ann Parasitol Hum Comp 1984 59 449 458 6508140

[B34] De Colmenares M Portús M Botet J Dobaño C Gallero M Wolff M Seguf G Identification of blood meals of *Phlebotomus perniciosus *(Diptera: Psychodidae) in Spain by a Competitive Enzyme-Linked Immunosorbent Assay Biotin/Avidin method J Med Entomol 1995 32 229 233 7616511 10.1093/jmedent/32.3.229

[B35] Quinnel RJ Dye C Shaw JJ Host preferences of the phlebotomine sandfly *Lutzomyia longipalpis *in Amazonian Brazil Med Vet Entomol 1992 6 195 200 10.1111/j.1365-2915.1992.tb00606.x 1421498

[B36] Bongiorno G Habluetzel A Khoury C Maroli M Host preference of phlebotomine sandflies at a hyperendemic focus of canine leishmaniasis in central Italy Acta Trop 2003 88 109 116 10.1016/S0001-706X(03)00190-6 14516922

[B37] Shaw JJ Lainson R Leishmaniasis in Brazil. II. Observations on enzootic rodent leishmaniasis in the lower Amazon region - the feeding habits of the vector, *Lutzomyia flaviscutellata *in reference to man, rodents and other animals Trans R Soc Trop Med Hyg 1968 62 396 405 10.1016/0035-9203(68)90091-6 5659233

[B38] Christensen HA Herrer A Panamanian *Lutzomyia *(Diptera: Psychodidae) host attraction profiles J Med Entomol 1980 17 522 528 7218267 10.1093/jmedent/17.6.522

[B39] Mutinga MJ Basimike M Kamau CC Mutero CM Epidemiology of leishmaniasis in Kenya, natural host preference of wild caught phlebotomine sandflies in Baringo District, Kenya East Afr Med J 1990 67 319 327 2390954

[B40] Killick-Kendrick R Killick-Kendrick M Focheux C Dereure J Puech M-P Cadiergues MC Protection of dogs from bites of phlebotomine sandflies by deltamethrin collars for control of canine leishmaniasis Med Vet Entomol 1997 11 105 111 10.1111/j.1365-2915.1997.tb00298.x 9226637

[B41] Maroli M Mizzoni V Siragusa C D'Orazi A Gradoni L Evidence for an impact on the incidence of canine leishmaniasis by the mass use of deltamethrin-impregnated dog-collars in southern Italy Med Vet Entomol 2001 15 358 363 10.1046/j.0269-283x.2001.00321.x 11776454

